# PFOS-elicited metabolic perturbation in liver and fatty acid metabolites in testis of adult mice

**DOI:** 10.3389/fendo.2023.1302965

**Published:** 2023-11-22

**Authors:** Wang Ka Lee, Thomas Ka Yam Lam, Hiu Ching Tang, Tsz Chun Ho, Hin Ting Wan, Chris Kong Chu Wong

**Affiliations:** ^1^Croucher Institute for Environmental Sciences, Department of Biology, Hong Kong Baptist University, Hong Kong, Hong Kong SAR, China; ^2^State Key Laboratory in Environmental and Biological Analysis, Hong Kong Baptist University, Hong Kong, Hong Kong SAR, China

**Keywords:** mass spectrophotometry imaging, hepatokines, lipogenesis, testosterone, fecundity

## Abstract

**Introduction:**

Multiple factors can contribute to sub-fecundity, including genetics, lifestyle, and environmental contaminants. PFASs are characterized as “forever chemicals” due to their ubiquitous contamination and their persistence in the environment, wildlife, and humans. Numerous studies have demonstrated that PFAS exposure adversely affects multiple bodily functions, including liver metabolism and gonadal function. It is unclear, however, how the disruption of hepatic fatty acid metabolism affects testicular function.

**Methods:**

In this study, male mice were administered 0.3 and 3 μg/g body weight of PFOS for 21 days.

**Results:**

Our data showed that PFOS exposure caused hepatic steatosis, as evidenced by significant increases in triglyceride levels, expression of ATP-citrate lyase, and fatty acid synthase, as well as fasting insulin levels. PFOS perturbed the expression levels of hepatokines, of which fibroblast growth factor-21 (*Fgf-21*), leukocyte cell-derived chemotaxin-2 (*Lect-2*), and retinol-binding protein-4 (*Rbp-4*) were significantly reduced, whereas angiopoietin-like 4 (*Angptl4*) was noticeably increased. While *Rbp-4 and Fgf-21* are known to contribute to spermatogenesis and testosterone synthesis. In PFOS-exposed groups, testicular ATP, and testosterone decreased significantly with a significant increase in the expression of peroxisome proliferator-activated receptor-coactivator 1α. Mass spectrophotometry imaging revealed the localization of PFOS in testes, along with significant increases in fatty acid metabolites. These included arachidonic acid, dihomo-α-linolenic acid, dihomo-γ-linolenic acid, oxidized ceramide, diacylglycerol, phosphatidylcholine, and phosphatidylethanolamine, which are associated with inflammation and post-testicular causes of infertility.

**Discussion:**

This study revealed potential links between PFOS-elicited changes in hepatic metabolism and their impacts on testicular biology. This study provides insights into alternative targets elicited by PFOS that can be used to develop diagnostic and therapeutic strategies for improving testicular dysfunction.

## Introduction

Lifestyle, environment, and genetic factors all contribute to sub-fecundity (1). Increasing evidence suggests that environmental chemical contaminants (e.g., heavy metals and anthropogenic chemicals) disrupt testicular physiology. Among different environmental chemical pollutants, per- and poly-fluoroalkyl substances (PFASs) are one of the prioritized family, known to perturb metabolic and reproductive health (2, 3). PFASs are coined as “forever chemicals” that contaminate public water systems, and linger in the environment, wildlife, and humans (4). The legacy PFAS, including PFOA, PFOS, PFNA, PFHxS share in common the lipophobic C-F chain and hydrophilic functional groups (5). PFASs are used in industrial and consumer products and have unique physicochemical properties, including heat and oil resistance, water repellence, and surfactant properties. They exhibited proteinophilic attraction toward albumin and various fatty acid binding proteins, resulting in their long biological half-lives and bioaccumulation in humans (6, 7). In recent decades, the direct effects of PFOS on testicular physiology have been studied in animal and cell culture models, revealing that it disrupted hormonal signaling in the testicles and perturbed the dynamics of tight junctional protein during spermatogenesis, which significantly affected males’ fertility and health (8–10). Even so, little is known about how the systemic impact of PFASs affects testicular function.

Over the past few years, patients seeking reproductive health care increasingly suffer from metabolic disorders, including obesity and insulin resistance (11–13). Since nutritional and hormonal factors influence energy metabolism and reproductive activity, to improve fertility rates, it is crucial to understand the underlying correlation of metabolic syndrome (14). The liver is a primary metabolic tissue that maintains energy and nutrient balance. Further, the liver metabolizes hormones and chemical contaminants, which may be degraded for excretion or bio-activated for more significant toxicity (15). Changes in metabolism affect hormone signals and nutrient flow, which can directly or indirectly affect gonadal function (16). One of the most notable effects of PFOS is the disruption of hepatic liver energy homeostasis, particularly fatty acid metabolism and the signaling of nuclear receptors (17–19). It is unclear how the disruption is related to the perturbing effect of testicular function. It was hypothesized that the disruption in fatty acid metabolism caused by PFASs could affect systemic energy metabolism and fatty acid metabolites in the testes. An integrated approach involving mass-spectrometry imaging, gene expression analysis, and biochemical testing were used in this study to investigate how PFAS affects the mammalian liver and testes. An analysis of the association between PFAS-induced metabolic perturbations and testicular dysfunction was conducted.

## Materials and methods

### Animals

Male CD-1 mice (8-10 weeks old) were kept in polypropylene cages at 23-24°C and 12 hours of light/dark cycle. The procedure for animal handling was followed according to guidelines and regulations approved by the animal ethics committee (REC/20-21/0234) of Hong Kong Baptist University. Perfluorooctane sulfonate (PFOS, CAS 1763-23-1, Sigma-Aldrich, 98% purity) was dissolved in dimethyl sulfoxide (Sigma-Aldrich) before mixing with corn oil. In the treatment regime, mice with body weights were randomly divided into three groups (control, low, or high-dose PFOS treatment groups) using a random number generator. The animals were provided access to food (LabDiet, 5001, Laboratory Rodent Diet) and water (in glass bottles). The exposed groups received either 0.3 or 3 μg/g of body weight (bw)/day of PFOS for 21 days by oral gavage (Cadence Science). Corn oil was administered to the control group. The low exposure dose is equivalent to human occupational exposure (20). Overnight fasting was performed on day 20. In the next morning, cervical dislocations were performed, and blood samples were drawn. Livers and testes were collected, snap-frozen in liquid nitrogen, and stored at -80°C.

### RNA extraction and real-time quantitative PCR (qPCR)

Total RNA was extracted from tissue samples using TRIzol reagent (Invitrogen) according to the manufacturer’s instruction. RNA concentration and quality were measured by BioDrop (Biochrom) and then reverse transcribed to cDNA using SuperScript VILO cDNA Synthesis Kit (Applied Biosystems). Gene expression was analyzed by real-time PCR using Fast SYBR™ Green Master Mix (Applied Biosystems) with StepOnePlus PCR system (Life Technologies) using gene-specific primers (**Supplementary Table 1A**). Relative expression was calculated by normalizing to *actin* using the 2^-ΔΔCt^ method. The specificity of the amplicon was verified using melting curve analysis and agarose gel electrophoresis.

### Western blot

Tissues were homogenized in RIPA buffer (50 mM Tris-HCl, pH 7.4, 150 mM NaCl, 2 mM EDTA, 0.1% SDS, and 1% NP-40) containing Halt™ Protease and Phosphatase Inhibitor Cocktail (Thermo Fisher Scientific). Tissue homogenates were then sonicated for 8 sec, 5 cycles on ice, followed by centrifugation at 13000 xg, at 4°C for 15min. Protein concentration was determined using the DC Protein Assay Kit II (BioRad). Protein samples were separated by SDS-PAGE and transferred to a PVDF membrane (BioRad). Membranes were blocked with 5% non-fat milk in PBST for 1 hr at room temperature, incubated with primary antibody (**Supplementary Table 1B**) overnight at 4°C and then incubated with the HRP-conjugated secondary antibody (BioRad) for 1 hr at room temperature. SuperSignal™ West Pico PLUS chemiluminescent substrate (Thermo Scientific) was used to develop the signals.

### Testosterone ELISA Kit, blood insulin, glucose *and ATP* determination

Adult male mouse were killed by cervical dislocation. Blood samples were collected and centrifuged at 1000 xg at 4°C for 10 min to collect sera, which were then stored at -80°C. Testosterone level in serum was measured using testosterone ELISA kit (Cayman Chemical). Briefly, 25 μl of serum sample or standard was mixed with 25 μl of testosterone AChE Tracer and 25 μl of testosterone ELISA antiserum in the mouse anti-rabbit IgG coated-well. The reaction mixture was discarded, and the wells were washed at room temperature on an orbital shaker (Thermo Fisher Scientific). Ellman’s Reagent (200μl) was added in each well and incubated for 90 minutes at room temperature in dark on the orbital shaker. Absorbance at 412 nm of each wells were measured using EnSight Multimode Plate Reader (PerkinElmer). The fasting blood glucose levels were measured using an Accu-check Glucometer (Roche, US). Serum was prepared by centrifugation of clotted blood at 1000 xg at 4°C for 10 min. Insulin level was measured with Ultrasensitive Insulin ELISA kit (10-1132-01, Mercodia, Sweden) according to the manufacturer’s instruction.

Testis samples were homogenized in luciferase cell culture lysis 5X reagent (Promega) and centrifuged at 13,000 ×g at 4°C for 15 min. The supernatants were collected for ATP measurement, using ATP Determination Kit (Invitrogen) according to the manufacturer’s instruction. Luminescence was measured by EnSight Multimode Plate Reader (PerkinElmer). ATP level was then normalized with the protein concentration of each sample using DC Protein Assay Kit II (BioRad).

### AFADESI-mass spectrophotometry imaging

Testes were isolated from adult male mice and snap-frozen in liquid nitrogen. The testis was mounted on a cryostat specimen chunk (Thermo Fisher Scientific, U.S.) and sliced at 14 μm in thickness using the Cryostar NX70 (Thermo Fisher Scientific). AFADESI-MSI (air-flow assisted desorption electrospray ionization) was applied to a frozen section mounted on a microscopic slide (Citotest, Jiangsu, China). The images were analyzed using an Orbitrap Exploris™ 120 mass spectrometer (Thermo Fisher Scientific, Bremen, Germany) and the AFAI-MSI image platform (Viktor, Beijing, China). A negative ion mode analysis was performed, with signals ranging from 100-1000m/z at a resolution of 60000. A spray solvent mixture of acetonitrile and dimethylformamide (3:1, v/v), was applied at 2 ml/min, with sprayer voltages at -2500V. The X- and Y-direction scanning speeds were 430 mm/s and 150 mm/s. A 140-psi gas flow and 350°C ion transfer tube temperature were used. MSConvert (Nature Biotechnology Commentary) and imzMLConverter (Thermo Fisher Scientific, U.S.) were used to convert MS data to mzML and imzML formats. SCiLSTM Lab (Bremen, Germany) was used to visualize data. The sections fixed in 4% PFA were then stained with hematoxylin.

### Statistical analysis

A statistical mean and standard deviation were used to present the data. The GraphPad Prism version 8.0 was used for statistical analyses. Students’-tests were used to evaluate the physiological and gene expression data. A p-value < 0.05 was considered statistically significant.

## Results

**Figure 1:**
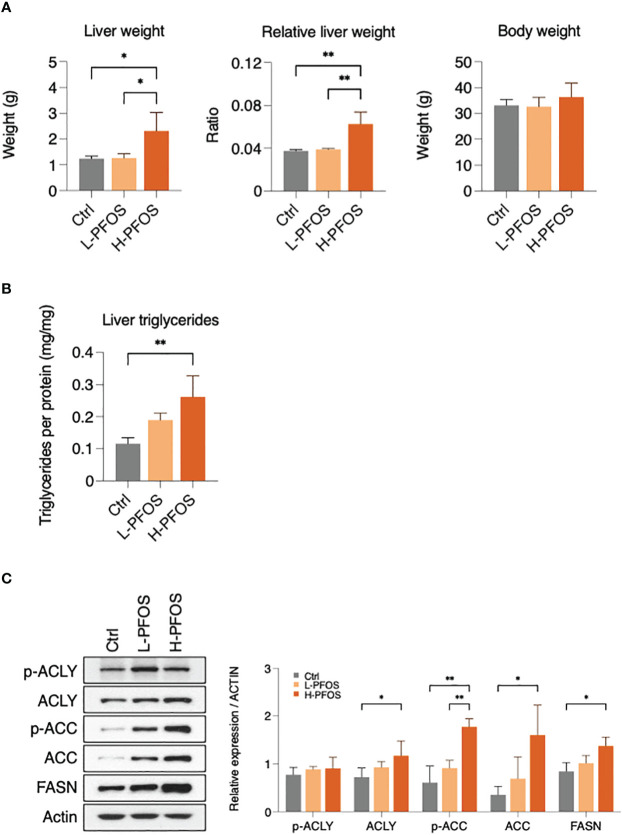
Effect of PFOS exposure on liver fat metabolism at day 21. Mice were administered 0.3 μg/g/day (L-PFOS) and 3 μg/g/day (H-PFOS) of PFOS by gavage for 21 days, and the liver was dissected and assessed. **(A)** The absolute and relative liver weights were significantly increased in H-PFOS mice compared to control and L-PFOS mice. **(B)** The triglyceride content was increased in H-PFOS compared to control, and **(C)** the protein expression of lipogenesis enzymes, including ATP citrate synthase (ACLY), phosphorylated ACLY (6X active), acetyl-CoA carboxylase (ACC), phosphorylated ACC (inactive form), and fatty acid synthase (FASN), were significantly upregulated in the PFOS-exposed groups. Actin served as the endogenous control. Graphs show the mean ± S.D. (*p<0.05, **p<0.01).

The exposure regime revealed a significant increase of liver weights and relative liver weights at the high-dose (3μg/g) of PFOS exposure while there was no noticeable effect on the changes of body weights among the control and PFOS treatment groups (**Figure 1A**). Additionally, hepatic triglyceride levels were found to be significantly increased at the high-dose exposure (**Figure 1B**). To underpin the underlying process of PFOS-elicited perturbation to hepatic lipid metabolism, western blot analysis of key metabolic enzymes for lipogenesis were conducted. The data revealed significant upregulation in the expression levels of ATP citrate lyase (ACLY), acetyl CoA carboxylase (ACC), phosphorylated ACC, and fatty acid synthase (FASN) at the high-dose PFOS-exposed groups (**Figure 1C**). The expression of ACLY and the ratio of pACC to ACC (**Supplementary Figure 1A**) did not change significantly.

**Figure 2:**
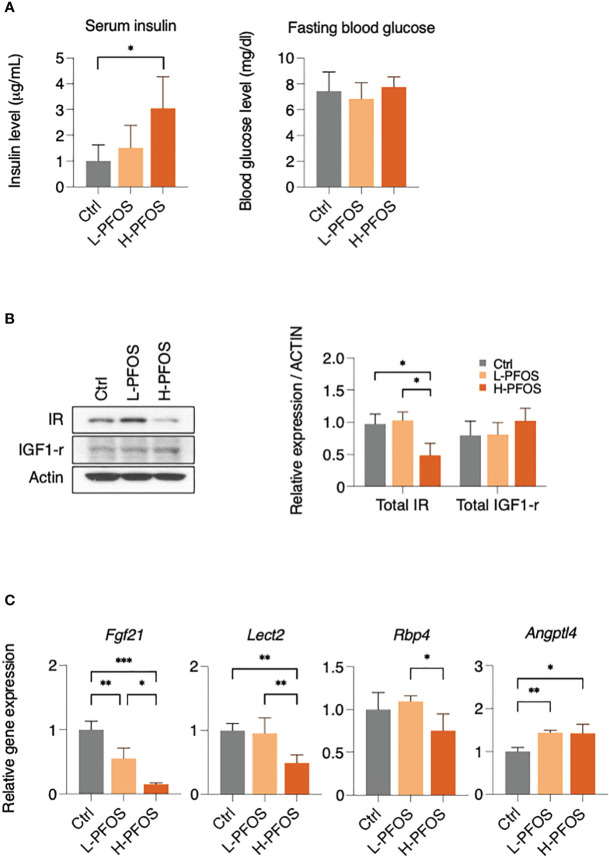
PFOS exposure perturb insulin signaling and expression of hepatokines in liver. **(A)** Serum insulin levels were significantly increased in H-PFOS compared to control, with no noticeable changes observed in fasting blood glucose levels. **(B)** The expression of insulin receptor (IR) was significantly downregulated. **(C)** A significant decrease in gene expression of the hepatokines, fibroblast growth factor 21 (*Fgf-21*) and leukocyte cell-derived chemotaxin 2 (*Lect-2*) were noted in PFOS-exposed groups, and a decrease of retinol binding protein 4 (*RBP-4*) was noted among L-PFOS and H-PFOS groups in livers. Conversely, the gene expression of angiopoietin-like 4 (*Angptl-4*) was increased in the H-PFOS compared to control. Actin served as the endogenous control. Graphs show the mean ± S.D. (*p<0.05, **p<0.01, ***p<0.001).

As the liver is the primary metabolic tissue, PFOS-induced disruptions in energy metabolism are likely to have a systemic effect. Fasting insulin and glucose levels were measured on day 21 of post-PFOS exposure. A significant increase in serum insulin levels was observed in the high-dose PFOS group, but no significant changes were observed in fasting blood glucose levels (**Figure 2A**). In western blot analysis, high-dose PFOS treatment significantly reduced hepatic expression levels of insulin receptor (IR) but not the insulin-like growth factor-1 receptor (IGF-1R) (**Figure 2B**). Considering the liver’s role in regulating systemic energy homeostasis, the expression levels of hepatokines, the major liver-to-tissue messengers that responds to perturbed energy metabolism, were investigated. Upon PFOS exposure, there was a dose-dependent reduction in the expression levels of the hepatokines, fibroblast growth factor-21 (*Fgf-21*), leukocyte cell derived chemotaxin-2 (*Lect-2*), retinol binding protein-4 (*Rbp-4*), but a significant induction of angiopoietin-like 4 (*Angptl4*) (**Figure 2C**). The expression levels of the other measured hepatokines, *Angptl-3*, *Angptl-6*, *Selenop*, and *Smoc-1* showed no noticeable differences (**Supplementary Figure 1B**).

**Figure 3:**
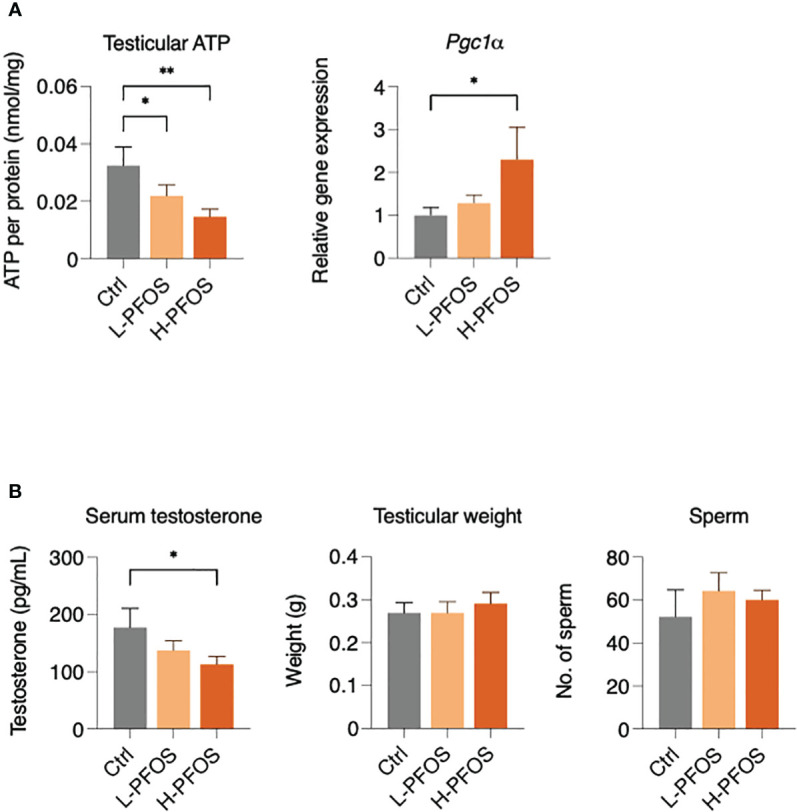
Impact of PFOS on testicular activity, energy metabolism and testosterone expression. **(A)** Testicular ATP levels were significantly decreased in PFOS-exposed groups. There was an increase in the gene expression of peroxisome proliferator-activated receptor gamma coactivator 1-α (*Pgc1α*) in H-PFOS groups compared to control. Actin served as the endogenous control. **(B)** Serum testosterone levels were significantly decreased in H-PFOS compared to control with no noticeable changes observed in testis weight and number of epididymal sperm counts. Graphs show the mean ± S.D. (*p<0.05, **p<0.01).

Due to PFOS’ effects on hepatic metabolism and serum insulin levels, it likely perturbed testicular functions. Western blot analysis showed that there were no significant changes in the expression levels of IR and phosphorylated IR in testes (**Supplementary Figure 1C**). However, PFOS-exposed groups showed a significant dose-dependent decrease of testicular ATP, associated with a significant increase in the expression of peroxisome proliferator-activated receptor γ coactivator 1α, *Pgc1α* (**Figure 3A**), a transcription coactivator in the regulation of cellular energy metabolism. Additionally, there was a significant reduction in testosterone levels in the high-dose PFOS exposed groups (**Figure 3B**, left panel). Yet, no significant change in testicular weight and epididymal sperm counts were noted (**Figure 3B**, right panel). Nonetheless, in testing the expression levels of endocrine and paracrine factors, there were no significant changes in the testicular gene expression of follicle-stimulating hormone receptor (*Fshr*) and luteinizing hormone receptor (*Lhr*) (**Supplementary Figure 2A**), growth hormone receptor (*Ghr*), insulin-like growth factor (*Igf*) and its receptor, *Igfr* (**Supplementary Figure 2B**), hepatocyte growth factor (*Hgf*) and its receptor, *Hgf-r* (**Supplementary Figure 2C**), and the steroidogenic enzymes (*Star, Cyp11a1, Cyp17a1, Hsd-3β, & Hsd-17β*) (**Supplementary Figure 3**) as compared with the control. Interesting, a significant reduction in the expression levels of Srd5α2 was noted in the high-dose group vs low-dose group.

**Figure 4:**
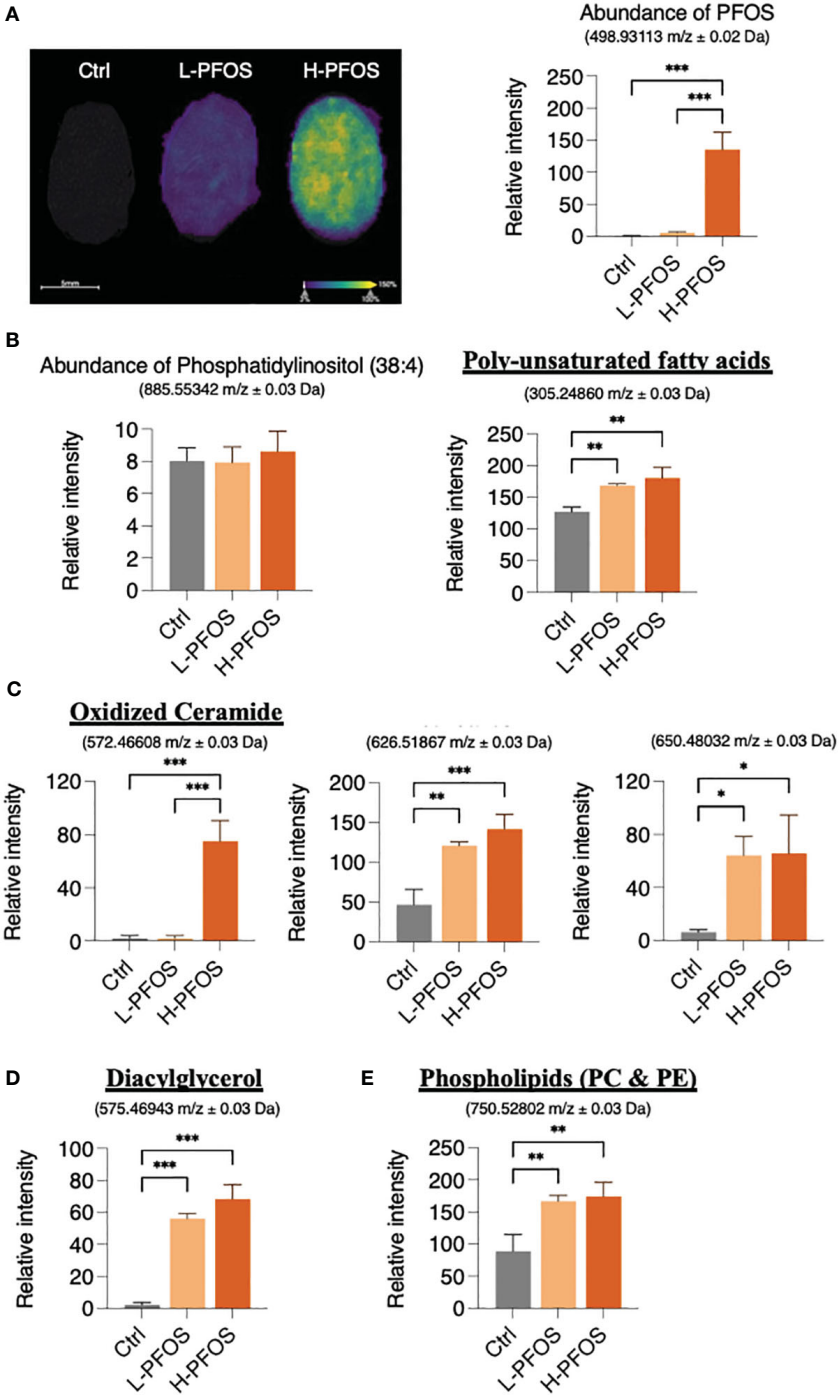
AFADESI-Mass Spectrophotometry Imaging of PFOS and metabolite distribution in testis. **(A)** Left panel: Ion image of PFOS distribution in testes with overlayed haematoxylin and eosin staining. Right panel: a corresponding graph shows the relative coneceantration of PFOS in the control, L-PFOS and H-PFOS samples. **(B)** Left panel: The levels of the phosphatidylinositol served for data normalization. Right panel: There was a significant increase in summated levels of the polyunsaturated fatty acids (eicosa-5, 8, 11-trienoic acid, eicosa-5, 11, 14-trienoic acid, dihomo-α-linolenic acid, dihomo-γ-linolenic acid), **(C)** oxidized ceramides (CER), **(D)** diacylglycerol (DAG) and **(E)** phosphatidylcholine (PC) & phosphatidylethanolamine (PE) in PFOS-exposed groups. Graphs show the mean ± S.D. (*p<0.05, **p<0.01, ***p<0.001).

PFOS is known to perturb fatty acid metabolism via PPARs and fatty acid-mimicry pathways. To unravel the possible impact of PFOS on fatty acid metabolites in testes, we examined PFOS uptake and its perturbation to lipid profiles using MS-imaging. **Figure 4A** showed a significant increase of PFOS levels in the testes of low- and high-dose exposed mice. MS-imaging data identified significant increases in the levels of the poly-unsaturated fatty acids, eicosa-5, 8, 11-trienoic acid (ETA), eicosa-5, 11, 14-trienoic acid (arachidonic acid, AA), dihomo-α-linolenic acid (DALA), and dihomo-γ-linolenic acid (DGLA) (**Figure 4B** right panel, **Supplementary Table 2**), oxidized ceramide (CER) (**Figure 4C**; **Supplementary Table 3**), diacylglycerol (DAG) (**Figure 4D**; **Supplementary Table 4**), and the phospholipids (phosphatidylcholine & phosphatidylethanolamine) (**Figure 4E**; **Supplementary Table 5**). The ion signal of phosphatidylinositol, which is a common and stable component of cell membranes (**Figure 4B**, left panel), was used for data normalization.

## Discussion

With hindsight, the effects of PFOS on both PPAR-dependent and PPAR-independent fatty acid mimicry pathways disrupt multiple metabolic pathways in multiple tissues, resulting in dyslipidemia, insulin resistance, and inflammation (21–24). This study showed that PFOS exposure at the high-dose (3μg/g b.w.) induced hepatic lipid accumulation which exhibited the early sign of non-alcoholic fatty liver disease (NAFLD). The observation is consistent with the previous studies, showing PFOS perturbed lipid metabolism and induced hepatotoxicity (22, 25). The hepatic liver accumulation was associated with the significant increase in the expression levels of ACLY and FASN. A combination of elevated expression of these enzyme activities promoted fatty acid synthesis from cytosolic acetyl-CoA. Despite this, the expression level of phosphorylated ACLY (pACLY) with reported 6-fold higher activity of ACLY (26) remained unchanged. The expression levels of both total ACC (active form) and pACC (inactive form) increased significantly with high-dose PFOS treatment. There was no significant change in the ratio of pACC to ACC, presumably no significant alteration in ACC activity. Nonetheless, it was unanticipated to see a significant increase in pACC with a functional outcome of hepatic lipogenesis in the group receiving high doses of PFOS. In retrospect, AMP-activated protein kinase (AMPK) is the major kinase known to phosphorylate ACC to promote fatty acid oxidation (27). In a recent study, PFOS-induced deranged hepatic metabolism stimulated both lipogenesis and lipid catabolism, as well as an activation of AMPK pathway (28). Therefore, in this study the observation of an increased phosphorylation of ACC might be associated with the dysregulation of hepatic metabolism induced by PFOS. The dysregulation was further demonstrated with a significant reduction in hepatic IR expression and a significant increase in blood insulin levels. This shows that PFOS perturbed insulin signaling in the liver and systemically.

Further illustrating the liver’s effect on bodily function, the secreted hepatic metabolic factors (hepatokines) were measured. In the PFOS-exposed groups, the expression levels of the four hepatokines (*Fgf-21*, *Lect-2*, *Rbp-4*, and *Angptl4*) were significantly altered. The four hepatokines were found to be associated with the progression of NAFLD (29–33). Apart from being a metabolic regulator, *Fgf-21* was also found to play roles in promoting spermatogenesis, protecting germ cells from diabetes-induced apoptosis (34, 35), and increasing sperm motility (36). *Lect-2* was linked to tissue inflammatory responses (37), coherent with the early sign of PFOS-elicited liver steatosis (22). Another perturbed hepatokine, *Rbp-4* regulated the physiological functions of testosterone receptors in the testicles, including the production of testosterone and spermatogenesis (38). In obese adolescents, *Rbp-4* expression was associated with gonadal functions (39). The decreased expression levels of *Fgf-21* and *Rbp4* imposed negative influence on testicular functions. In fact, fatty liver disease has also been linked to impaired testicular function (40, 41). There were reports indicating an association between NAFLD and low blood levels of testosterone, which caused accumulation of visceral adipose tissues, elevated free fatty acid levels, inflammation, and increased insulin resistance (42, 43). The observations suggest that systemic metabolism and gonadal function are mutually interdependent.

In testes, metabolic perturbation was evidenced with significant reduction in ATP levels and the increased expression of *Pgc-1α*, which was reported to protect against oxidative stress and energy metabolism dysfunction in the testes (44). Previous studies had shown that men with fatty liver disease exhibited lower levels of testosterone and sperm count (41, 45, 46). In this study, our data only showed the reduction of blood testosterone levels at the high-dose group. In our previous studies, a higher dose of PFOS exposure (5 μg/g b.w. for 21 days) in mice, caused significant reduction in testosterone levels, sperm count, and sperm swimming activities (8, 9, 47). To decipher the underlying process of the reduced testosterone levels in the high-dose group, the analysis of the steroidogenic enzymes did not reveal noticeable changes, except a significant reduction of Srd5α2 (steroid 5α-reductase 2, a membrane enzyme catalyzes testosterone to dihydrotestosterone). From a pharmacological perspective, an inhibition of Srd5α2 activity might be linked to an increase in serum testosterone levels (48). This may explain an increase in serum testosterone in the high-dose group of our study. In order to further investigate the impact of PFOS on testicular physiology, we focused on the notorious action of PFOS in perturbing fatty acid homeostasis, which may negatively impact testicular functions (49, 50).

The PFOS exposure increased the testicular levels of the poly-unsaturated fatty acids (ETA and AA), the important metabolites in regulating gonadal functions (51, 52). This observation is related to our previous study showing an increase of hydroxyeicosatetraenoic acids in neonatal testes upon PFOS exposure (49). Additionally, PFOS-induced an increase of the poly-unsaturated fatty acids (DGLA and DGLA), which were reported to be directly associated with the sign of tissue inflammation (53, 54). Moreover, our data showed a significant increase in oxidized ceramides and DAG, the important messengers for spermatogenesis, and apoptosis (55, 56). Furthermore, significant increases in total phospholipids, especially phosphatidylcholine, and phosphatidylethanolamine, were associated with the post-testicular causes of infertility (57). Overall, the data suggest that PFOS disrupted fatty acid metabolites’ homeostasis, altering the fatty acid signaling pathway in testes.

This study aimed to better understand the pleiotropic effects of PFOS on tissue functions. As shown by our data, PFOS affects hepatic lipid metabolism, hepatokine expression, blood insulin, testosterone levels, and testicular fatty acid metabolism. This study provided a mechanistic link between disrupted lipid metabolism and perturbed testicular physiology. These findings may help identify PFOS disregard targets and develop targeted interventions to restore and protect testicular function in mammals exposed to these pollutants. As a result of this study, we have gained a better understanding of the pathology, the molecular mechanisms, and the biochemical changes resulting from PFAS-induced testicular toxicity.

## Data availability statement

The original contributions presented in the study are included in the article/**Supplementary Material**. Further inquiries can be directed to the corresponding authors.

## Ethics statement

The procedure for animal handling was followed according to guidelines and regulations approved by the animal ethics committee (REC/20-21/0234) of Hong Kong Baptist University. The study was conducted in accordance with the local legislation and institutional requirements.

## Author contributions

WL: Data curation, Formal Analysis, Investigation, Writing – review & editing. TL: Formal Analysis, Investigation, Methodology, Software, Writing – review & editing. HT: Investigation, Methodology, Writing – review & editing. TH: Data curation, Investigation, Writing – review & editing. HW: Conceptualization, Investigation, Methodology, Project administration, Supervision, Writing – review & editing. CW: Conceptualization, Funding acquisition, Supervision, Writing – original draft, Writing – review & editing.
