# Supplementary material for: PFOS-elicited metabolic perturbation in liver and fatty acid metabolites in testis of adult mice
Source: Front Endocrinol (Lausanne). 2023 Nov 22;14:1302965. doi: 10.3389/fendo.2023.1302965 (PMC10703039; doi:10.3389/fendo.2023.1302965)
Supplement: Supplementary file 1 [file Presentation_1.pptx]

## Slide 1
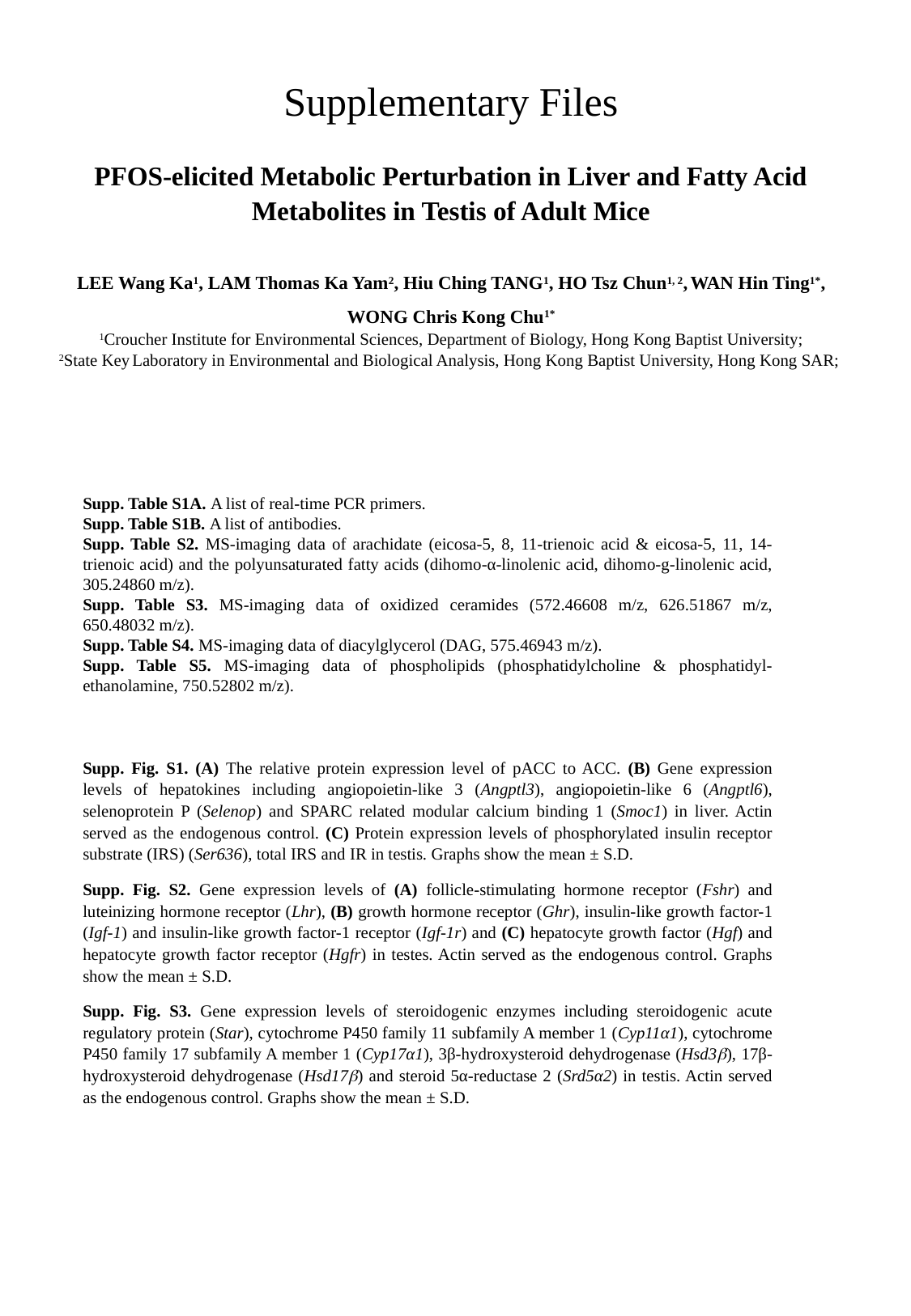

Supplementary Files
PFOS-elicited Metabolic Perturbation in Liver and Fatty Acid Metabolites in Testis of Adult Mice
LEE Wang Ka1, LAM Thomas Ka Yam2, Hiu Ching TANG1, HO Tsz Chun1, 2, WAN Hin Ting1*, WONG Chris Kong Chu1*
1Croucher Institute for Environmental Sciences, Department of Biology, Hong Kong Baptist University;
2State Key Laboratory in Environmental and Biological Analysis, Hong Kong Baptist University, Hong Kong SAR;
Supp. Table S1A. A list of real-time PCR primers.
Supp. Table S1B. A list of antibodies.
Supp. Table S2. MS-imaging data of arachidate (eicosa-5, 8, 11-trienoic acid & eicosa-5, 11, 14-trienoic acid) and the polyunsaturated fatty acids (dihomo-α-linolenic acid, dihomo-g-linolenic acid, 305.24860 m/z).
Supp. Table S3. MS-imaging data of oxidized ceramides (572.46608 m/z, 626.51867 m/z, 650.48032 m/z).
Supp. Table S4. MS-imaging data of diacylglycerol (DAG, 575.46943 m/z).
Supp. Table S5. MS-imaging data of phospholipids (phosphatidylcholine & phosphatidyl-ethanolamine, 750.52802 m/z).
Supp. Fig. S1. (A) The relative protein expression level of pACC to ACC. (B) Gene expression levels of hepatokines including angiopoietin-like 3 (Angptl3), angiopoietin-like 6 (Angptl6), selenoprotein P (Selenop) and SPARC related modular calcium binding 1 (Smoc1) in liver. Actin served as the endogenous control. (C) Protein expression levels of phosphorylated insulin receptor substrate (IRS) (Ser636), total IRS and IR in testis. Graphs show the mean ± S.D.
Supp. Fig. S2. Gene expression levels of (A) follicle-stimulating hormone receptor (Fshr) and luteinizing hormone receptor (Lhr), (B) growth hormone receptor (Ghr), insulin-like growth factor-1 (Igf-1) and insulin-like growth factor-1 receptor (Igf-1r) and (C) hepatocyte growth factor (Hgf) and hepatocyte growth factor receptor (Hgfr) in testes. Actin served as the endogenous control. Graphs show the mean ± S.D.
Supp. Fig. S3. Gene expression levels of steroidogenic enzymes including steroidogenic acute regulatory protein (Star), cytochrome P450 family 11 subfamily A member 1 (Cyp11α1), cytochrome P450 family 17 subfamily A member 1 (Cyp17α1), 3β-hydroxysteroid dehydrogenase (Hsd3β), 17β-hydroxysteroid dehydrogenase (Hsd17β) and steroid 5α-reductase 2 (Srd5α2) in testis. Actin served as the endogenous control. Graphs show the mean ± S.D.

## Slide 2
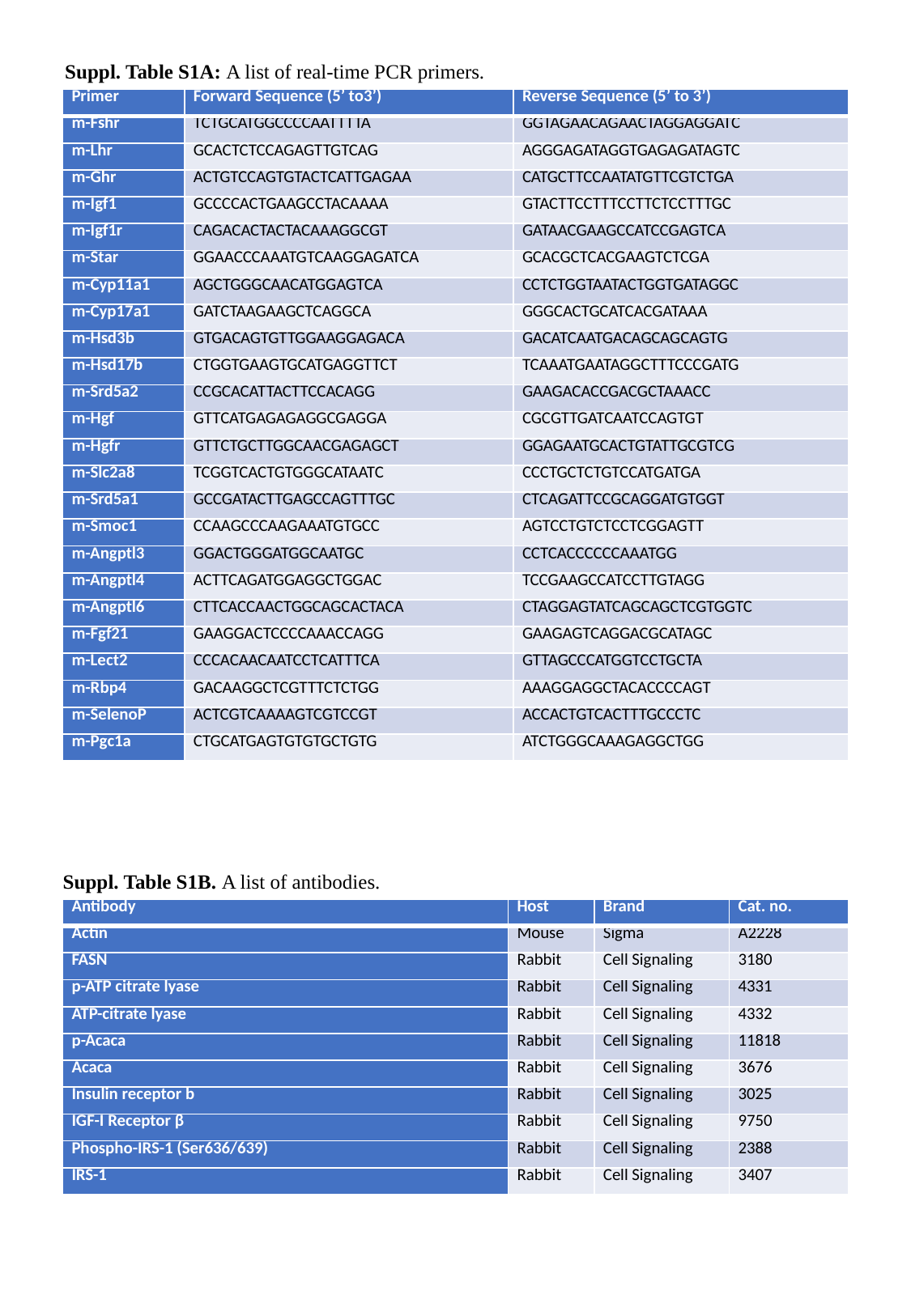

Suppl. Table S1A: A list of real-time PCR primers.
| Primer | Forward Sequence (5’ to3’) | Reverse Sequence (5’ to 3’) |
| --- | --- | --- |
| m-Fshr | TCTGCATGGCCCCAATTTTA | GGTAGAACAGAACTAGGAGGATC |
| m-Lhr | GCACTCTCCAGAGTTGTCAG | AGGGAGATAGGTGAGAGATAGTC |
| m-Ghr | ACTGTCCAGTGTACTCATTGAGAA | CATGCTTCCAATATGTTCGTCTGA |
| m-Igf1 | GCCCCACTGAAGCCTACAAAA | GTACTTCCTTTCCTTCTCCTTTGC |
| m-Igf1r | CAGACACTACTACAAAGGCGT | GATAACGAAGCCATCCGAGTCA |
| m-Star | GGAACCCAAATGTCAAGGAGATCA | GCACGCTCACGAAGTCTCGA |
| m-Cyp11a1 | AGCTGGGCAACATGGAGTCA | CCTCTGGTAATACTGGTGATAGGC |
| m-Cyp17a1 | GATCTAAGAAGCTCAGGCA | GGGCACTGCATCACGATAAA |
| m-Hsd3b | GTGACAGTGTTGGAAGGAGACA | GACATCAATGACAGCAGCAGTG |
| m-Hsd17b | CTGGTGAAGTGCATGAGGTTCT | TCAAATGAATAGGCTTTCCCGATG |
| m-Srd5a2 | CCGCACATTACTTCCACAGG | GAAGACACCGACGCTAAACC |
| m-Hgf | GTTCATGAGAGAGGCGAGGA | CGCGTTGATCAATCCAGTGT |
| m-Hgfr | GTTCTGCTTGGCAACGAGAGCT | GGAGAATGCACTGTATTGCGTCG |
| m-Slc2a8 | TCGGTCACTGTGGGCATAATC | CCCTGCTCTGTCCATGATGA |
| m-Srd5a1 | GCCGATACTTGAGCCAGTTTGC | CTCAGATTCCGCAGGATGTGGT |
| m-Smoc1 | CCAAGCCCAAGAAATGTGCC | AGTCCTGTCTCCTCGGAGTT |
| m-Angptl3 | GGACTGGGATGGCAATGC | CCTCACCCCCCAAATGG |
| m-Angptl4 | ACTTCAGATGGAGGCTGGAC | TCCGAAGCCATCCTTGTAGG |
| m-Angptl6 | CTTCACCAACTGGCAGCACTACA | CTAGGAGTATCAGCAGCTCGTGGTC |
| m-Fgf21 | GAAGGACTCCCCAAACCAGG | GAAGAGTCAGGACGCATAGC |
| m-Lect2 | CCCACAACAATCCTCATTTCA | GTTAGCCCATGGTCCTGCTA |
| m-Rbp4 | GACAAGGCTCGTTTCTCTGG | AAAGGAGGCTACACCCCAGT |
| m-SelenoP | ACTCGTCAAAAGTCGTCCGT | ACCACTGTCACTTTGCCCTC |
| m-Pgc1a | CTGCATGAGTGTGTGCTGTG | ATCTGGGCAAAGAGGCTGG |
Suppl. Table S1B. A list of antibodies.
| Antibody | Host | Brand | Cat. no. |
| --- | --- | --- | --- |
| Actin | Mouse | Sigma | A2228 |
| FASN | Rabbit | Cell Signaling | 3180 |
| p-ATP citrate lyase | Rabbit | Cell Signaling | 4331 |
| ATP-citrate lyase | Rabbit | Cell Signaling | 4332 |
| p-Acaca | Rabbit | Cell Signaling | 11818 |
| Acaca | Rabbit | Cell Signaling | 3676 |
| Insulin receptor b | Rabbit | Cell Signaling | 3025 |
| IGF-I Receptor β | Rabbit | Cell Signaling | 9750 |
| Phospho-IRS-1 (Ser636/639) | Rabbit | Cell Signaling | 2388 |
| IRS-1 | Rabbit | Cell Signaling | 3407 |

## Slide 3
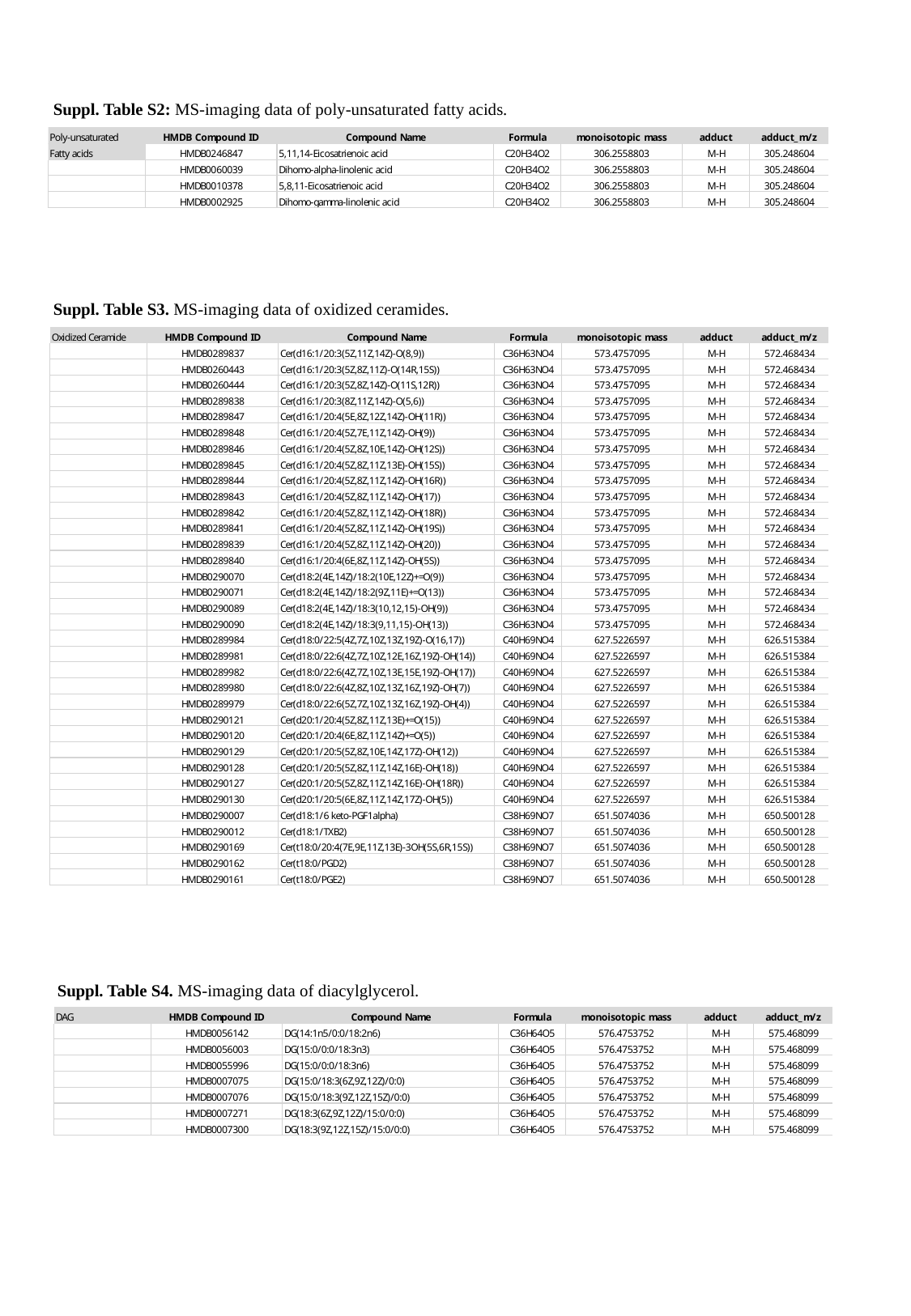

Suppl. Table S2: MS-imaging data of poly-unsaturated fatty acids.
Suppl. Table S3. MS-imaging data of oxidized ceramides.
Suppl. Table S4. MS-imaging data of diacylglycerol.

## Slide 4
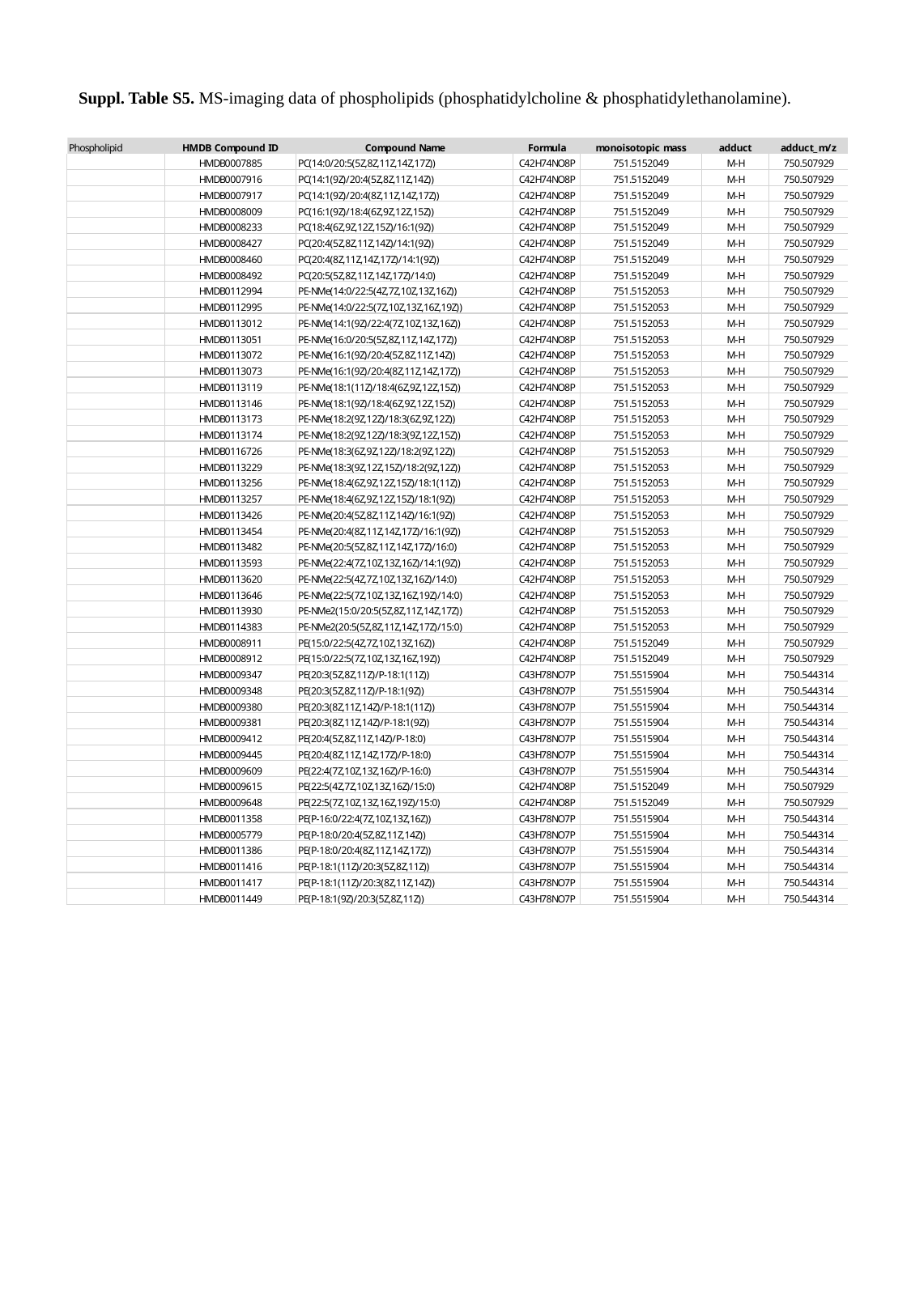

Suppl. Table S5. MS-imaging data of phospholipids (phosphatidylcholine & phosphatidylethanolamine).

## Slide 5
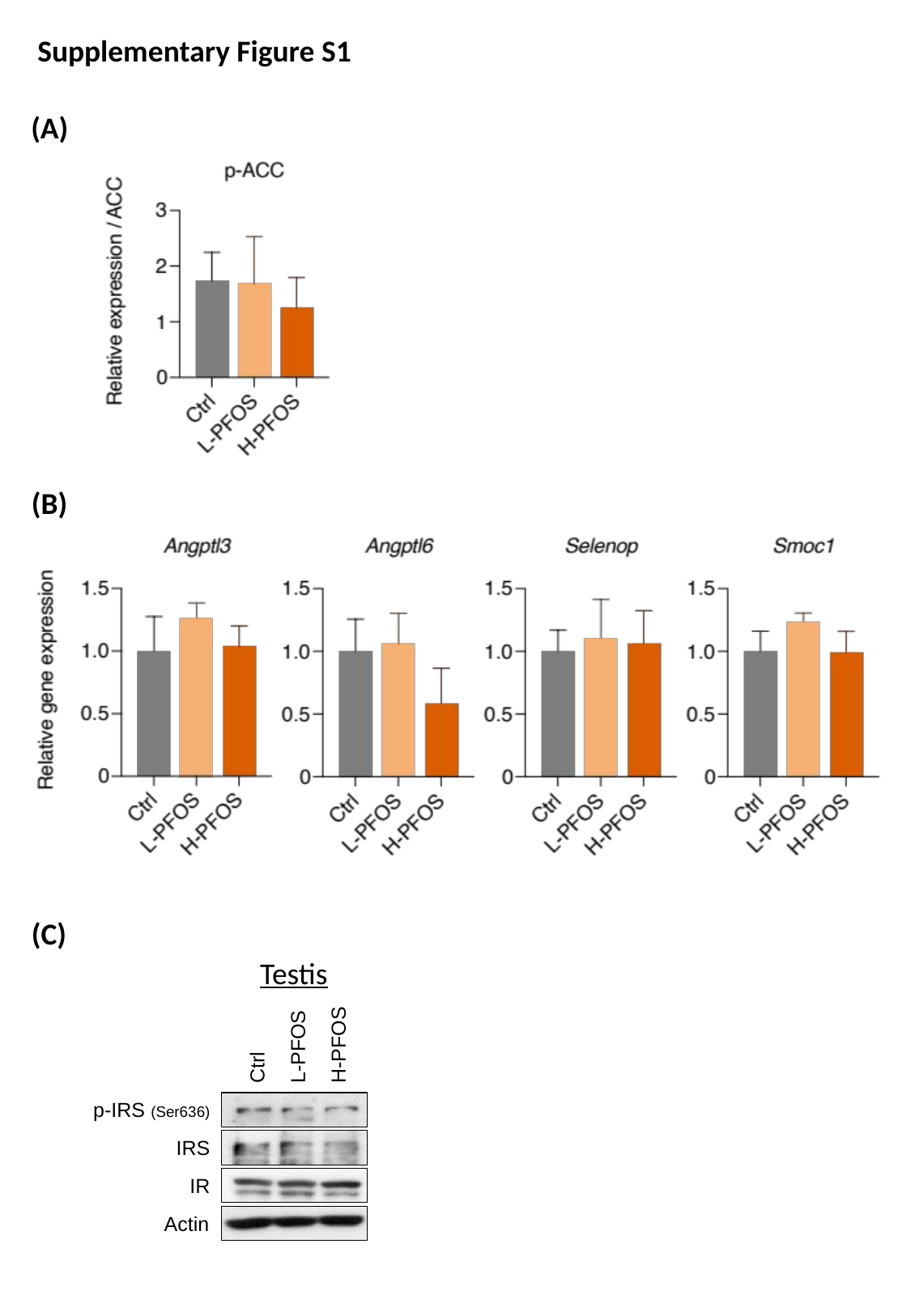

Supplementary Figure S1
(A)
(B)
(C)
H-PFOS
L-PFOS
Ctrl
p-IRS (Ser636)
IRS
IR
Actin
Testis

## Slide 6
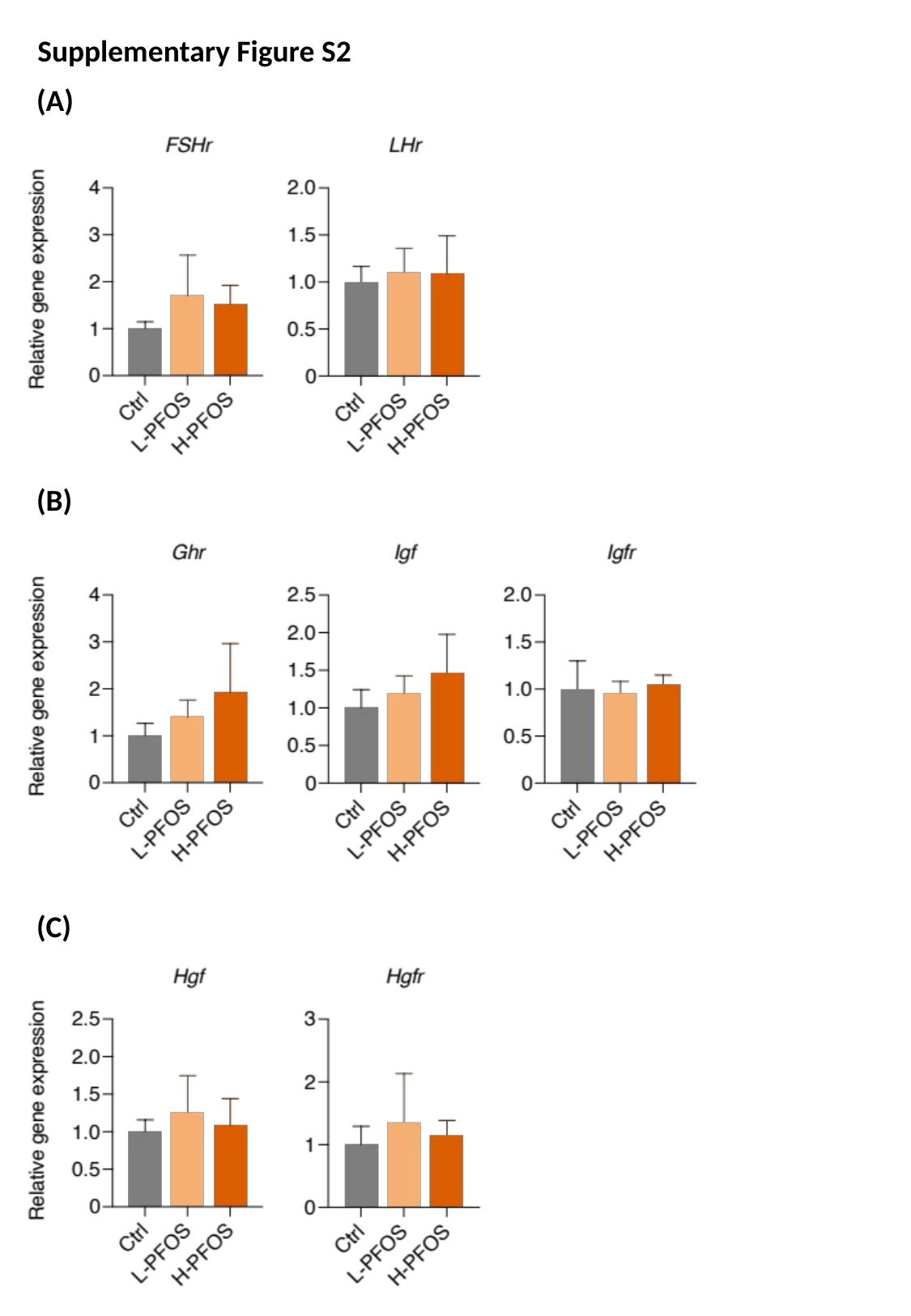

Supplementary Figure S2
(A)
(B)
(C)

## Slide 7
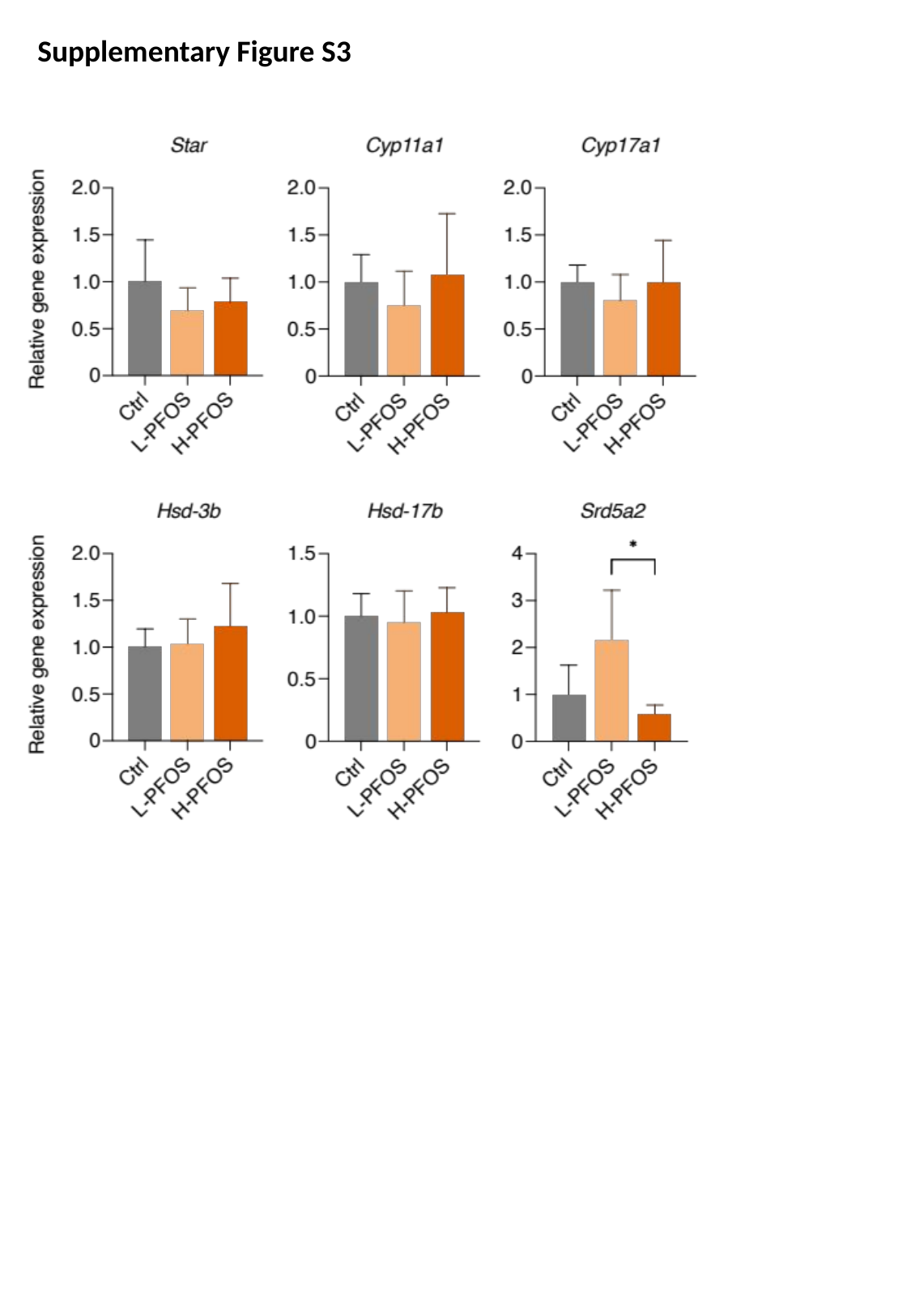

Supplementary Figure S3
